# Long non‐coding RNA *SUMO1P3* promotes hepatocellular carcinoma progression through activating Wnt/β‐catenin signalling pathway by targeting *miR‐320a*

**DOI:** 10.1111/jcmm.14977

**Published:** 2020-01-22

**Authors:** Songsong Wu, Sheng Chen, Ning Lin, Jianchuan Yang

**Affiliations:** ^1^ Department of Ultrasonography Fujian Provincial Hospital Shengli Clinical Medical College of Fujian Medical University Fuzhou China

**Keywords:** hepatocellular carcinoma, long non‐coding RNA, microRNAs, *SUMO1P3*, Wnt/β‐catenin pathway

## Abstract

In this study, we aimed to investigate expression profile of long non‐coding RNA (lncRNA) *SUMO1P3,* and its role and molecular mechanisms in the progression of hepatocellular carcinoma (HCC).

The expression of *SUMO1P3 *in HCC tissues and cells was detected using quantitative real‐time polymerase chain reaction (qRT‐PCR). The chi‐squared test was used to estimate the relationship between *SUMO1P3* levels and clinical characteristics of HCC cases. Cellular biological behaviours were investigated using MTT, transwell assays and wound healing assay. Bioinformatics and dual‐luciferase reporter assays were performed to identify potential target of *SUMO1P3 *in HCC. Additionally, protein analysis was carried out using Western blot.

The expression of *SUMO1P3* was significantly higher in HCC tissues and cells than in non‐cancerous specimens and normal cells (*P* < .01). Moreover, its up‐regulation was closely correlated with lymph node metastasis (*P* = .027) and TNM stage (*P* = .019). *SUMO1P3* knockdown inhibited the proliferation, migration and invasion of HCC cells. *MiR‐320a* was a potential target of *SUMO1P3*, and its expression was negatively regulated by *SUMO1P3 *in HCC *SUMO1P3* could activate Wnt/β‐catenin pathway, which was mediated by *miR‐320a*.

Elevated expression of *SUMO1P3* predicts malignant progression among HCC patients. *SUMO1P3* enhances Wnt/β‐catenin pathway through sponging *miR‐320a*, thus contributing to aggressive progression of HCC.

## 1 INTRODUCTION

Hepatocellular carcinoma (HCC) is a frequently diagnosed fatal malignancy, posing a great threat to human health around the world, especially in China.^1^, ^2^ The morbidity of HCC is closely correlated with the infections of hepatitis B virus (HBV) and hepatitis C virus (HCV), smoking, alcohol abuse, etc^3^ In addition, cirrhosis, diabetes and obesity may also increase the risk of HCC.^4^ At present, therapeutic strategies for HCC mainly include liver transplantation, resection, local ablative therapies, adjuvant chemotherapy and immune treatment.^5^ Even though these treatments could improve the outcomes of HCC patients, long‐time prognosis of the disease is still unsatisfactory, due to high rates of recurrence and distant metastasis.^6^ Therefore, it is crucial to identify key factors that drive the tumorigenesis of HCC.

Long non‐coding RNAs (lncRNAs) are a class of endogenous RNAs with a length of more than 200 nucleotides.^7^ Although lncRNAs hold no or limited ability to code proteins, they may take part in various biological processes through regulating gene expression at transcriptional and post‐transcriptional levels.^8^ lncRNAs could interact with RNA and DNA through base pairing to form regulatory netweb with DNA, protein and RNA, thus playing important roles in physiological and pathological conditions.^9^, ^10^ In tumorigenesis, lncRNAs could exert promoting or repressing effects, or both.^11^ In HCC, expression patterns of lncRNA show close association with cell proliferation, apoptosis, invasion and metastasis, suggesting their possibilities to be employed as diagnostic and prognostic biomarkers, and therapeutic targets.^12^

Small ubiquitin‐like modifier (SUMO) 1 pseudogene 3 (*SUMO1P3*) is a pseudogene and belongs to a separate class of lncRNAs.^13^ The up‐regulation of lncRNA *SUMO1P3* has been observed in several human cancers, such as breast cancer,^13^ gastric cancer 14 and bladder cancer.^15^ In HCC, the study performed by Zhou et al^16^ demonstrated that the knockdown of *SUMO1P3* could inhibit cell proliferation, colony formation and invasion abilities. However, molecular mechanisms underlying the function of *SUMO1P3 *in HCC progression remained poorly understood.

In this study, we investigated expression patterns of lncRNA *SUMO1P3 *in HCC tissues and cells. Moreover, we estimated the association of *SUMO1P3* expression with clinical characteristics of HCC patients. In addition, cell experiments were designed to explore the mechanisms of *SUMO1P3 *in HCC progression.

## 2 MATERIALS AND METHODS

### 2.1 Patients and tissue collection

The study was performed in Fujian Provincial Hospital, Shengli Clinical Medical College of Fujian Medical University. The patients who were newly diagnosed with HCC via pathological examinations in this hospital were included in our study. None of the patients had received any treatments, either surgery, radiotherapy, chemotherapy or immune therapy. The study was approved by the Ethics Committee of the hospital. Written informed consent was obtained from all patients or their families before surgery.

Hepatocellular carcinoma tissues and adjacent normal ones were collected in surgery. The tissues were immediately frozen in liquid nitrogen, and then stored at −80°C. Clinical characteristics of the patients were collected from their medical records. A total of 104 HCC patients including 60 males (57.7%) and 44 females (42.3%) were enrolled in our study, with an average age of 61.25 ± 10.36 years. Of the patients, 42 (40.4%) had smoking history, while 60 (57.7%) showed evidences for drinking. Additionally, 60 patients (57.7%) exhibited tumour size more than 3 cm, and lymph node metastasis was observed in 37 patients (35.6%). According to TNM (tumour node metastasis) staging, 59 (56.7%) patients were at stages I‐II, and 45 (43.3%) at stages III‐IV. Clinical information of the patients was summarized in Table 1.

**Table 1:** The association of *SUMO1P3* expression with clinical characteristics of HCC patients

Characteristics	N (n = 104, %)	*SUMO1P3* low expression (n = 46, %)	*SUMO1P3* high expression (n = 58, %)	*P‐*values
Age (y)				
≥60	64 (61.5)	27 (48.7)	37 (63.8)	.596
<60	40 (38.5)	19 (42.3)	21 (36.2)	
Gender				
Male	60 (57.7)	26 (56.5)	34 (58.6)	.850
Female	44 (42.3)	20 (43.5)	24 (42.4)	
Smoking				
Yes	42 (40.4)	18 (39.1)	24 (41.4)	.816
No	62 (59.6)	28 (60.9)	34 (58.6)	
Drinking				
Yes	60 (57.7)	28 (60.9)	32 (55.2)	.559
No	44 (42.3)	18 (39.1)	26 (44.8)	
Tumour size (cm)				
≤3	60 (57.7)	30 (65.2)	30 (51.7)	.167
>3	44 (42.3)	16 (34.8)	28 (48.3)	
Lymph node metastasis				
Yes	37 (35.6)	11 (23.9)	26 (44.8)	.027
No	67 (64.4)	35 (76.1)	32 (22.2)	
TNM stage				
I‐II	59 (56.7)	32 (69.6)	27 (46.6)	.019
III‐IV	45 (43.3)	14 (30.4)	31 (53.4)	

### 2.2 Cell line and culture

In our study, in vitro experiments were carried out using human HCC cell line HepG2 (code: TCHu 72) and normal hepatocyte cell line THLE3 (code: GNHu40). Both of the two cell lines were bought from the Cell Bank of the Chinese Academic of Science (CBP600232; Shanghai, China). The cell lines were cultured using Roswell Park Memorial Institute 1640 (RPMI 1640) medium supplemented with 10% FBS, 100 U/mL penicillin and 100 μg/mL streptomycin. Cell mediums were maintained in an incubator containing 5% CO_2_ at 37°C.

### 2.3 RNA extraction and quantitative analysis

RNA template was extracted from collected tissue and cell samples using TRIzol reagent (Invitrogen, Thermo Fisher Scientific, Inc) according to the manufacturer's instruction. Then, RNA sample was used for the synthesis of the first‐strand cDNA, and the reaction was implemented using PrimeScript RT Reagent Kit (Takara). Quantitative analysis for the genes was carried out using quantitative real‐time polymerase chain reaction (qRT‐PCR) method. Specific primer sequences were as follows: *GAPDH* forward: 5′‐TGCACCACCAACTGCTTAGC‐3′; reverse: 5′‐GGCATGGACTGTGGTCATGAG‐3′; *SUMO1P3* forward: 5′‐ACTGGGAATGGAGGAAGA‐3′; reverse: 5′‐TGAGAAAGGATTGAGGGAAAAG‐3′; *U6* forward: 5′‐CTCGCTTCGGCAGCACA‐3′; reverse: 5′‐AACGCTTCACGAATTTGCGT‐3′; *miR* *‐320a* forward: 5′‐GGGCTAAAAGCTGGGTTGA‐3′; reverse: 5′‐CAGTGCGTGTCGTGGAGT‐3′. *GAPDH* was employed as an internal control for mRNA detection, while *U6* acted as a control for miRNA detection. 2^−ΔΔCt^ equation was used to calculate relative expression of the genes. Each test was repeated three times.

### 2.4 Cell transfection

siRNA targeting *SUMO1P3* (si‐*SUMO1P3*) and corresponding negative control (si‐NC) were designed and synthesized by HANBIO company. The recombinants were transfected to HCC cell line HepG2 using Lipofectamine^®^ 2000 reagent (Invitrogen, Thermo Fisher Scientific, Inc) according to the instructions. Transfected cells were incubated at 37°C with 5% CO_2_ for 48 hours. Then, the cells were harvested, and qRT‐PCR method was used to investigate the expression of *SUMO1P3* to estimate transfection efficacy.

### 2.5 Cell proliferation

Cell proliferation ability was estimated through MTT assay using MTT Cell Proliferation and Cytotoxicity Assay Kit (Sangon Biotech). In brief, cells were seeded to a 96‐well plate with a density of ×10^5^ cells/mL. Then, the cells were incubated at 37°C with 5% CO_2_. At an interval of one day, 20 μL MTT was supplemented into cell medium and incubated for an additional 4 hours. Then, 150 μL DMSO was added and incubated at dark for 10 minutes to stop reaction. Subsequently, a microplate reader (TECAN) was used to detect the absorbance of the cell medium at 490 nm to estimate cell proliferation. Each test was carried out in triplicate.

### 2.6 Cell migration and invasion

The effects of *SUMO1P3* expression on the motility of HCC cells were evaluated using Transwell assay (8.0 µm pore size, Costar). In migration analysis, 500 μL RPMI 1640 medium was added into the upper chamber, while the lower chamber was coated with 500 μL RPMI 1640 medium supplemented with 10% FBS. Two hundred micro litre cell suspension with a density of 5 × 10^4^ cells/mL was seeded into the upper chamber, and then, the chamber was incubated at 37°C with 5% CO_2_. Forty eight hours later, the cells in the lower chamber were stained using crystal violet and counted under an inverted microscope (IX31; Olympus Corporation). For each sample, five random files were selected. For invasion analysis, Matrigel (Corning Glass Works) was added into the upper chamber, and the procedures were carried out in accordance with the Migration analysis. Each test had three repeats.

### 2.7 Wound healing assay

To test migration results from transwell assay, we conducted wound healing assay. HepG2 cells were seeded into 6‐well plates (4 × 10^5 ^cells/well), and 2% FBS‐supplemented medium was added to avoid cell proliferation before incubation at 37°C for 24 hours. si‐NC (NR) and si‐*SUMO1P3* were transfected into HepG2 cells. Then, freshly changed 2% FBS‐supplemented medium was added after the medium was removed, and wounds were created with a sterile 200‐µL pipette tip in each well. Wound healing was monitored and photographed at 0, 24, 48 and 72 hours.

### 2.8 Dual‐luciferase reporter assay

Bioinformatics and dual‐luciferase reporter systems were used to confirm potential targeted genes of *SUMO1P3 *in HCC. Then, potential targeted miRNAs of *SUMO1P3* were identified on starBase (http:// http://starbase.sysu.edu.cn/) and miRanda (http://www. microrna.org/microrna/home.do). The luciferase reporter plasmids containing *SUMO1P3* wild type (wt) or *SUMO1P3* mutant type (mut) were constructed, and cotransfected with *miR* *‐320a* mimic or mimic NC into HCC cell line HepG2. Cell transfection was performed using Lipofectamine 3000 (Invitrogen, Thermo Fisher Scientific, Inc), and then, the cells were incubated at 37°C with 5% CO_2_. Forty eight hours later, luciferase activity of the cells was detected via Dual‐Luciferase Reporter Assay System (Promega Corporation). Renilla luciferase activity was normalized to firefly luciferase activity.

### 2.9 Western blot analysis

Protein expression was detected using Western blot analysis in our study. Protein samples were isolated from cell and tissue specimens using RIPA Lysis and Extraction Buffer (Thermo Scientific). Quantified analysis of protein sample was performed through BCA method, which was carried out using a BCA Protein Assay Kit (Thermo Scientific). Then, equal amount of protein samples was separated using 10% SDS‐PAGE analysis. Later, targeted proteins were transfected onto polyvinylidene fluoride membrane (0.45 µm pore size; EMD Millipore), and blocked employing 5% skimmed milk for 90 minutes at room temperature. Subsequently, the membranes were separated using specific primary antibodies, including anti‐β‐catenin antibody (1:5000, Abcam), anti‐C‐myc antibody (1:1000, Abcam), anti‐cyclin D1 antibody (1:10 000, Abcam) and anti‐GAPDH antibody (1:10 000, Abcam). GAPDH was employed as a loading control. Next, the membranes were incubated with secondary anti‐rabbit IgG antibody (1:2000, Abcam) at room temperature for 2 hours. Finally, band grey was detected by a ECL substrate reagent kit (GE Healthcare) on a Gel Doc XR imaging system (Bio‐Rad).

### 2.10 Statistical analysis

All data calculations were performed using SPSS 18.0 software (SPSS, Inc), and figures were plotted applying GraphPad Prism version 5.0 software (GraphPad). Continuous variables were shown as average ± standard deviation (SD), and their differences between two groups were analysed through Student's *t* test. Categorical data were recorded as case number, and their comparison between two groups was carried out using the chi‐squared test. All tests were two‐tailed, and *P‐*values <.05 meant the statistical significance of the results.

## 3 RESULTS

### 3.1 Up‐regulation of *SUMO1P3 *in HCC

**Figure 1:**
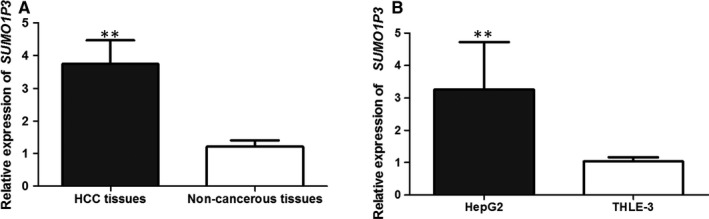
The expression of *SUMO1P3* was significantly enhanced in HCC tissues (A) and cell line (B). **P < .01

qRT‐PCR was used to investigate the expression of *SUMO1P3* mRNA in HCC tissues and cells. The results displayed in Figure 1 showed that the levels of *SUMO1P3* were higher in HCC tissues than in adjacent normal ones (*P* < .01, Figure 1A). Moreover, compared to normal hepatic cells, the expression of *SUMO1P3* was obviously enhanced in HCC cell line HepG2 (*P* < .01, Figure 1B).

### 3.2 Association of *SUMO1P3* mRNA with clinical characteristics of HCC patients

According to the mean expression of *SUMO1P3* mRNA in HCC tissues, the included HCC patients were divided into high (n = 58) and low (n = 46) expression groups. The chi‐squared test was applied to estimate the relationship between *SUMO1P3* expression and clinical information of HCC patients. We found that the levels of *SUMO1P3* mRNA were positively correlated with lymph node metastasis (*P* = .027) and TNM stage (*P* = .019). However, the expression of *SUMO1P3* was not influenced by HCC patients' age, gender, smoking, drinking habits or tumour size (*P* > .05 for all) (Table 1).

### 3.3 Knockdown of *SUMO1P3* suppressed HCC cell proliferation, migration and invasion

**Figure 2:**
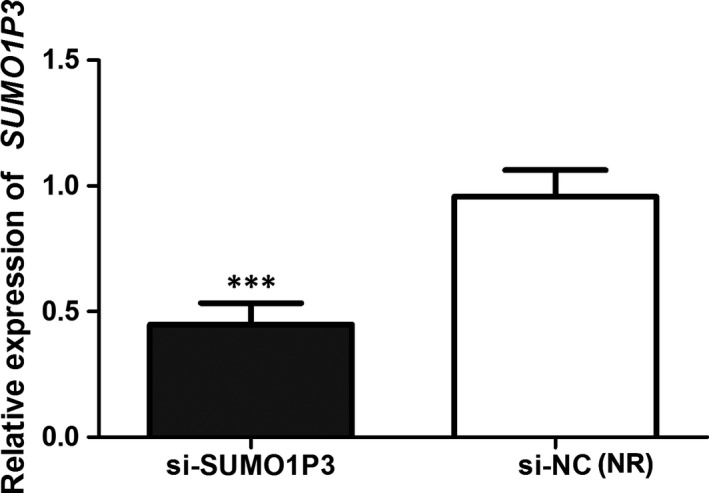
The transfection with si‐*SUMO1P3* leads to obvious down‐regulation of *SUMO1P3 *in HepG2 cells. ***P < .001; NR: negative reference; si‐*SUMO1P3*: HepG2 cells transfected by si‐*SUMO1P3* plasmid; si‐NC: HepG2 cells transfected by negative control plasmid, acted as NR

In order to investigate the function of *SUMO1P3 *in HCC progression, HepG2 cells were transfected by si‐*SUMO1P3* vector to inhibit the expression of *SUMO1P3*. qRT‐PCR analysis suggested that the levels of *SUMO1P3* were significantly decreased after the transfection with si‐*SUMO1P3* (*P* < .001, Figure 2).

**Figure 3:**
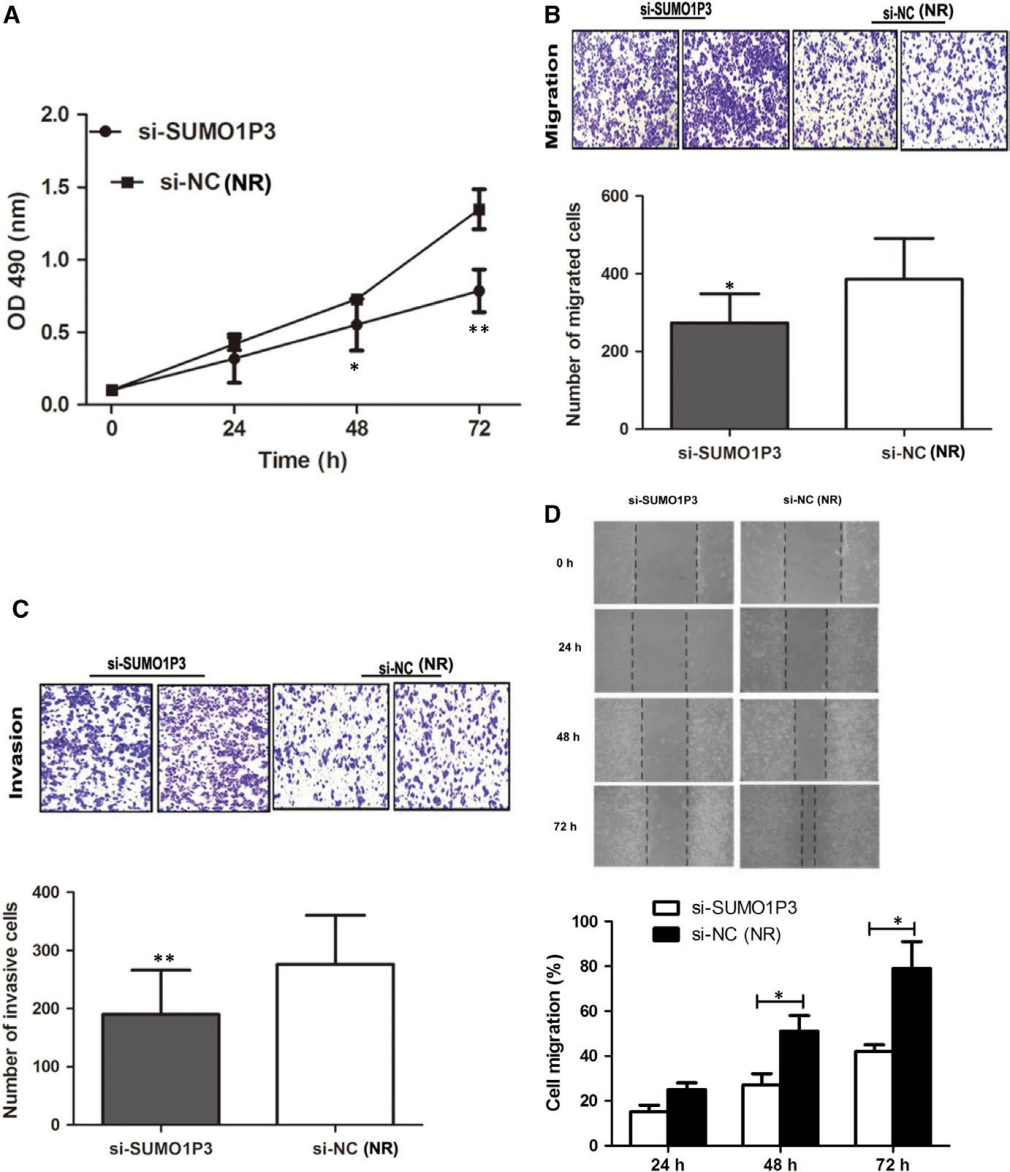
Knockdown of *SUMO1P3* obviously suppresses HCC cell proliferation (A), migration (B, C) and invasion (D, E). *P < .05, **P < .01; NR: negative reference; si‐*SUMO1P3*: HepG2 cells transfected by si‐*SUMO1P3* plasmid; si‐NC: HepG2 cells transfected by negative control plasmid, acted as NR

MTT and transwell assays were used to detect cell proliferation, migration and invasion of the transfected cells, separately. The results demonstrated that the transfection with si‐*SUMO1P3* could significantly inhibit cell proliferation (*P* < .05, Figure 3A), migration (*P* < .05, Figure 3B) and invasion (*P* < .01, Figure 3C). Moreover, wound healing assay also verified results on migration (*P* < .05, Figure 3D). Knockdown of *SUMO1P3* might inhibit the proliferation, migration and invasion abilities of HCC cells.

### 3.4 *SUMO1P3 *acted as sponge of *miR‐320a* in HCC

**Figure 4:**
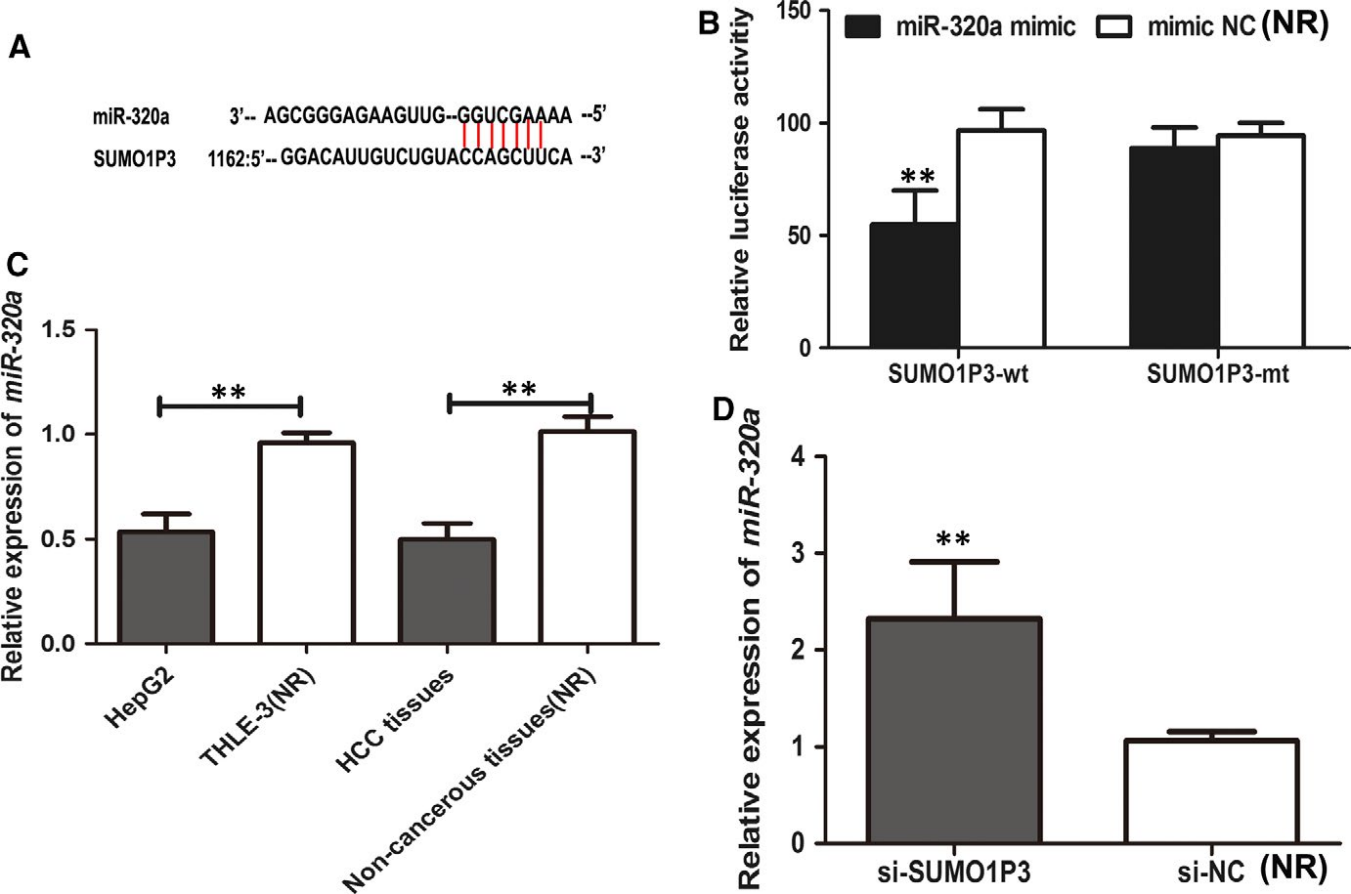
Bioinformatics analysis confirmed that the 3′‐end of *SUMO1P3* had complementary sequences of *miR‐320a* (A). Dual‐luciferase reporter analysis was performed to ascertain the relationship between *SUMO1P3* and *miR‐320a*. The presence of *miR‐320a* and *SUMO1P3* decreased luciferase activity of HepG2 cells, but *miR‐320a* presence did not influence luciferase activity of HepG2 cells transfected by SUMO1P3‐mt (B). The expression of *miR‐320a* was decreased in HCC cells (HepG2) compared to normal hepatic cell line THLE‐3 (C), and the levels of *miR‐320a* were obviously lower in HCC tissues than in non‐malignant ones. But the knockdown of *SUMO1P3* enhanced the expression of *miR‐320a* (D). **P < .01; NR: negative reference; si‐*SUMO1P3*: HepG2 cells transfected by si‐*SUMO1P3* plasmid; si‐NC: HepG2 cells transfected by negative control plasmid, acted as NR

Bioinformatics analysis demonstrated that the 3′‐end of *SUMO1P3* possessed complementary sequences of *miR‐320a* (Figure 4A). Thus, dual‐luciferase reporter assay was performed to verify whether *miR‐320a* was a potential target of *SUMO1P3 *in HCC. The results displayed in Figure 4B suggested that HCC cells cotransfected by *SUMO1P3* wt and *miR‐320a* mimic exhibited obviously low luciferase activity than those cotransfected by *SUMO1P3* wt and mimic NC (*P* < .01), while the cotransfection with *miR‐320a* mimic and *SUMO1P3* mt did not significantly affect luciferase activity of the cells, compared to the control (*P* > .05). *MiR‐320a* might bind to *SUMO1P3*.

In addition, we examined the expression of *miR‐320a* in HCC tissues and HepG2 cells. The results demonstrated that the expression of *miR‐320a* was significantly decreased in HepG2 cells, compared to normal hepatic cells. Meanwhile, HCC tissues showed obviously reduced expression of *miR‐320a* in comparison with non‐cancerous ones (*P* < .01, Figure 4C). Moreover, the knockdown of *SUMO1P3* could obviously enhance the expression of *miR‐320a* in HepG2 cells (*P* < .01, Figure 4D). *SUMO1P3* could sponge the expression of *miR‐320a*.

### 3.5 *SUMO1P3* suppressed Wnt/β‐catenin pathway in HCC

**Figure 5:**
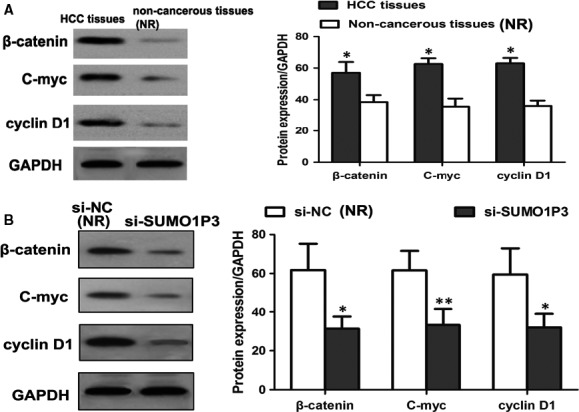
Compared to non‐cancerous ones, HCC tissues showed significantly increased expressions of β‐catenin, C‐myc and cyclin D1 proteins (A). The inhibition of *SUMO1P3 *in HepG2 cells resulted in the down‐regulation of β‐catenin, C‐myc and cyclin D1 proteins, revealing the inactivation of Wnt/β‐catenin pathway (B). *P < .05, **P < .01; NR: negative reference; si‐*SUMO1P3*: HepG2 cells transfected by si‐*SUMO1P3* plasmid; si‐NC: HepG2 cells transfected by negative control plasmid, acted as NR

The study carried out by Lu et al^17^ reported that β‐catenin was a potential targeted gene of *miR‐320a* in HCC and that *miR‐320a* could regulate the activity of Wnt/β‐catenin pathway. Given the relationship between *SUMO1P3* and *miR‐320a*, we investigated regulatory function of *SUMO1P3* on Wnt/β‐catenin pathway in HCC. Western blot analysis suggested that the protein levels of β‐catenin, C‐myc and cyclin D1 were higher in HCC tissues than in non‐cancerous ones (*P* < .05 for all, Figure 5A). Moreover, the knockdown of *SUMO1P3* could remarkably suppress the expression of β‐catenin, C‐myc and cyclin D1 proteins (*P* < .05 for all, Figure 5B), revealing the inactivation of Wnt/β‐catenin pathway.

### 3.6 Oncogenic function of *SUMO1P3 *was mediated by *miR‐320a*

**Figure 6:**
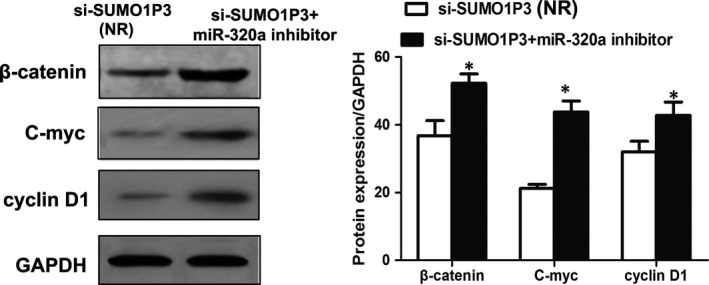
The cotransfection with si‐*SUMO1P3* and *miR‐320a* inhibitor leads to the up‐regulation of β‐catenin, anti‐C‐myc and cyclin D1 proteins. *P < .05, **P < .01; NR: negative reference; si‐*SUMO1P3*: HepG2 cells transfected by si‐*SUMO1P3* plasmid, which acted as NR; si‐*SUMO1P3* + *miR‐320a* inhibitor: HepG2 cells cotransfected by si‐*SUMO1P3* and *miR‐320a* inhibitor

Additional experiments were designed to ascertain whether the function of *SUMO1P3 *in HCC progression was mediated by *miR‐320a*. HepG2 cells were cotransfected by si‐*SUMO1P3* and *miR‐320a* inhibitor, and cells transfected by si‐*SUMO1P3* vector were employed as controls. Western blot analysis suggested that compared to the controls, the cotransfection with si‐*SUMO1P3* and *miR‐320a* inhibitor led to up‐regulated β‐catenin, C‐myc and cyclin D1 proteins (*P* < .05 for all; Figure 6).

**Figure 7:**
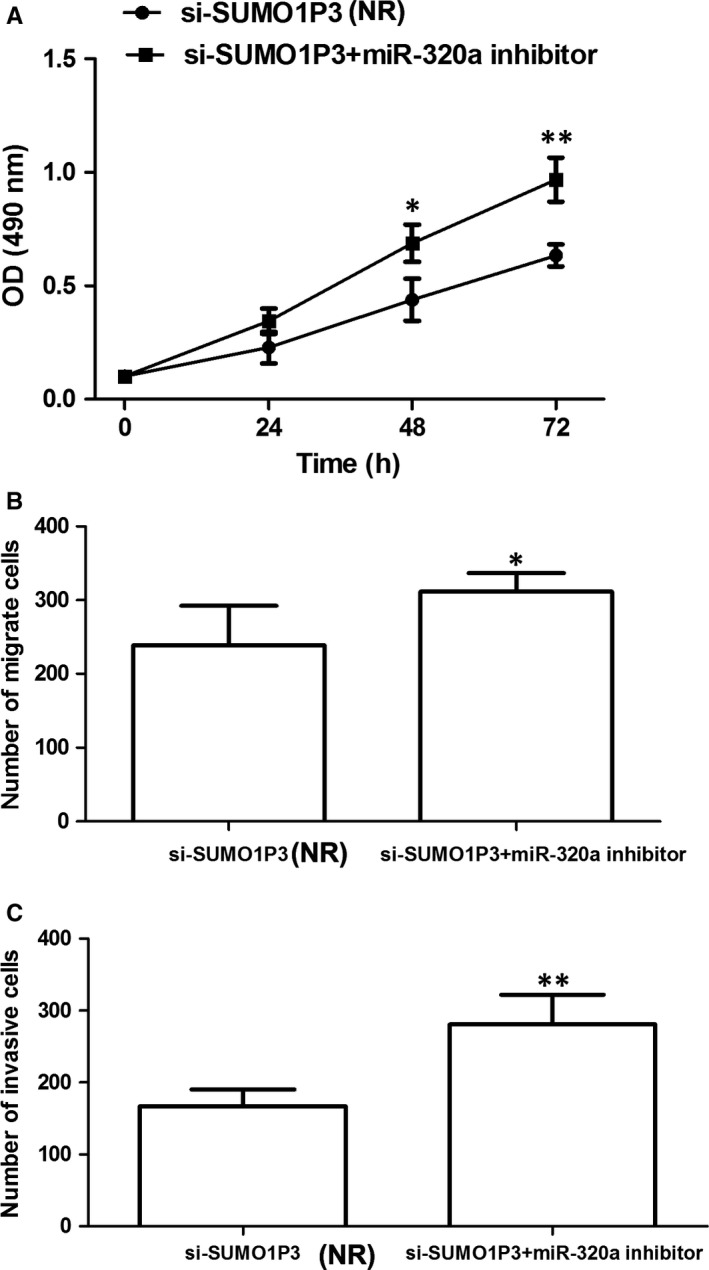
The presence of *miR‐320a* inhibitor significantly promoted HCC cell proliferation (A), migration (B) and invasion (C). *P < .05, **P < .01; NR: negative reference; si‐*SUMO1P3*: HepG2 cells transfected by si‐*SUMO1P3* plasmid, which acted as NR; si‐*SUMO1P3* + *miR‐320a* inhibitor: HepG2 cells cotransfected by si‐*SUMO1P3* and *miR‐320a* inhibitor

In addition, MTT assay suggested that the presence of *miR‐320a* inhibitor significantly promoted HCC cell proliferation, and the migration and invasion ability were confirmed by transwell analysis (*P* < .05 for all; Figure 7). All data revealed that promoting function of *SUMO1P3 *in HCC progression was dependent on *miR‐320a*. *MiR‐320a* had the ability to reverse oncogenic function of *SUMO1P3 *in HCC.

## 4 DISCUSSION

The tumorigenesis of HCC is regulated by the interactions between genetic and epigenetic mutations.^18^ Gene silencing mediated by lncRNAs is a prevalent epigenetic change in tumorigenesis.^19^ In HCC, a variety of lncRNAs have been confirmed to be involved in cancer progression. For example, Zhu et al^20^ found that lncRNA NEAT1 interacting with *miR‐384* contributed to HCC progression via promoting cell proliferation, migration and invasion. Wang et al^21^ demonstrated that lncRNA DGCR5 exerted suppressive function in HCC progression through miR‐346/KLF14 axis. lncRNAs are involved in various biological processes in HCC tumorigenesis, and may be employed as therapeutic targets, thus improving clinical outcomes of the patients. In this study, we investigated clinical significance and related molecular mechanisms of lncRNA *SUMO1P3 *in HCC progression. The results indicated that lncRNA *SUMO1P3* might be an oncogene in HCC through enhancing cell proliferation, migration and invasion, and its up‐regulation showed close association with lymph node metastasis and TNM stage. *SUMO1P3* could activate Wnt/β‐catenin pathway that is mediated by *miR‐320a*, thus contributing to malignant progression of HCC.

Expression levels of lncRNA *SUMO1P3* were significantly higher in HCC tissues and cells than in non‐cancerous specimens. Moreover, its up‐regulation predicted positive lymph node metastasis and advanced TNM stages. The overexpression of *SUMO1P3* might contribute to malignant progression of HCC. In subsequent cell experiments, we found that the knockdown of *SUMO1P3* obviously suppressed the proliferation, migration and invasion abilities of HCC cells. The conclusion was consistent with that from published article. Zhou et al^16^ reported that HCC patients with high expression of *SUMO1P3* were more likely entering advanced TNM stages, while its inhibition might suppress cell proliferation and motility, and promote cell apoptosis. All data revealed that *SUMO1P3* acted as an oncogene in HCC.

Despite the lack of protein‐coding ability, lncRNAs could regulate gene expression at both transcriptional and post‐transcriptional levels.^8^ At post‐transcriptional level, interaction between lncRNAs and microRNAs (miRNAs) is an important pathway to regulate gene expression.^22^ In our study, we found that the 3′ end of lncRNA *SUMO1P3* had complementary sequence of *miR‐320a*. Moreover, subsequent dual‐luciferase reporter assay demonstrated that *miR‐320a* was a potential target of *SUMO1P3*. *SUMO1P3* could negatively regulate the expression of *miR‐320a* in HCC. The up‐regulation of *SUMO1P3* resulted in the down‐regulation of *miR‐320a*, which could promote HCC cell proliferation and invasion, thus contributing to malignant growth and metastasis.^23^, ^24^, ^25^ Our results were in line with those from published articles. The interaction between *SUMO1P3* and *miR‐320a* was also confirmed in breast cancer.^13^ *SUMO1P3* interacting with *miR‐320a* played an important role in HCC progression.

Growing evidences have demonstrated that *SUMO1P3* promotes tumorigenesis through regulating epithelial‐mesenchymal transition (EMT) signalling pathway and angiogenesis.^26^, ^27^ Molecular mechanisms of *SUMO1P3 *in HCC progression were also explored in our study. The study carried out by Lu et al^17^ suggested that *miR‐320a* could suppress HCC progression based on its inhibition on Wnt/β‐catenin pathway. β‐Catenin was a direct target of *miR‐320a* in HCC. In the current study, we found that the knockdown of *SUMO1P3* led to the inactivation of Wnt/β‐catenin signalling pathway. Moreover, regulatory function of *SUMO1P3* on Wnt/β‐catenin pathway was depended on *miR‐320a*. The absence of *miR‐320a* could reverse anti‐tumour action caused by the knockdown of *SUMO1P3*. Taken together, *SUMO1P3* contributed to malignant progression of HCC through activating Wnt/β‐catenin pathway via negatively regulating *miR‐320a*. However, the study carried out by Xie et al^25^ demonstrated that *miR‐320a* played anti‐tumour action in HCC through directly targeting c‐Myc. In addition, HMGB1 was confirmed to be a targeted gene of *miR‐320a* in HCC.^23^ All evidences proved that *SUMO1P3* might influence multiple signalling pathways through its targeted genes in HCC. However, due to limited study period, regulatory netweb of *SUMO1P3 *in HCC was not completely explored in our study. Besides, the sample size was relatively small. Clinical significance of *SUMO1P3 *in HCC requires further investigations with extended sample size. Additionally, experiments in our study only proved that the expression levels of *SUMO1P3* could influence the oncogenicity of HCC cells, and whether the overexpression of *SUMO1P3* could endow normal hepatic cells with oncogenic ability remained unclear. Lastly, the absence of animal experiments might limit statistical power of our results. Therefore, further researches are in urgent need to address the above issues.

In conclusion, lncRNA *SUMO1P3* is up‐regulated in HCC specimens and positively correlated with metastasis and tumour stage. *SUMO1P3* has the ability to promote the proliferation, invasion and migration of HCC cells. In HCC, *SUMO1P3* could activate *miR‐320a*–mediated Wnt/β‐catenin signalling pathway, thus contributing to aggressive progression of the disease.

## CONFLICT OF INTEREST

None.

## AUTHORS' CONTRIBUTIONS

SW and SC conceived and designed the experiments, analysed the data and wrote the paper. NL and JY performed the experiments. All authors read and approved the final manuscript.

## Data Availability

All data generated or analysed during this study are included in this article.
